# Resonant Scattering in Proximity‐Coupled Graphene/Superconducting Mo_2_C Heterostructures

**DOI:** 10.1002/advs.202201343

**Published:** 2022-05-23

**Authors:** Meng Hao, Chuan Xu, Cheng Wang, Zhen Liu, Su Sun, Zhibo Liu, Hui‐Ming Cheng, Wencai Ren, Ning Kang

**Affiliations:** ^1^ Key Laboratory for the Physics and Chemistry of Nanodevices and Center for Carbon‐based Electronics, School of Electronics Peking University Beijing 100871 China; ^2^ Shenyang National Laboratory for Materials Science Institute of Metal Research Chinese Academy of Sciences Shenyang 110016 China

**Keywords:** 2D materials, graphene heterostructure, proximity effect, resonant scattering

## Abstract

The realization of high‐quality heterostructures or hybrids of graphene and superconductor is crucial for exploring various novel quantum phenomena and devices engineering. Here, the electronic transport on directly grown high‐quality graphene/Mo_2_C vertical heterostructures with clean and sharp interface is comprehensively investigated. Owing to the strong interface coupling, the graphene layer feels an effective confinement potential well imposed by two‐dimensional (2D) Mo_2_C crystal. Employing cross junction device geometry, a series of resonance‐like magnetoresistance peaks are observed at low temperatures. The temperature and gate voltage dependences of the observed resonance peaks give evidence for geometric resonance of electron cyclotron orbits with the formed potential well. Moreover, it is found that both the amplitude of resonance peaks and conductance fluctuation exhibit different temperature‐dependent behaviors below the superconducting transition temperature of 2D Mo_2_C, indicating the correlation of quantum fluctuations and superconductivity. This study offers a promising route toward integrating graphene with 2D superconducting materials, and establishes a new way to investigate the interplay of massless Dirac fermion and superconductivity based on graphene/2D superconductor vertical heterostructures.

## Graphical abstract

Directly grown graphene/Mo_2_C vertical heterostructures provide an ideal platform for exploring the interplay of massless Dirac fermion and superconductivity. This low‐temperature transport reveals the geometric resonance by the interaction of the carriers with the boundaries of the potential well formed at the graphene/Mo_2_C interface, and the potential well can be effectively modified through superconducting proximity effect in heterostructures.

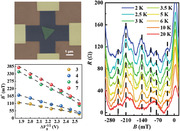

## 1 Introduction

In recent years, intense attention has been devoted to the study of graphene and other 2D crystals, which not only lead to the emergence of exotic electronic states, but also serve as atomically thin building blocks for the development of novel devices with new functionalities and promising applications.^[^ ^1^, ^2^, ^3^ ^]^ In particular, the superconductor‐graphene heterostructures or hybrid devices have been explored to engineer unique interfacial quantum phenomena, such as ballistic proximity superconductivity, spectral Andreev reflections, chiral Andreev edge states, and topological superconductivity.^[^ ^4^, ^5^, ^6^, ^7^ ^]^ For these hybrid superconducting devices, a highly transparent superconductor‐graphene junction is critically important. Thus improving the quality of junction device and creating a clean and strong coupled interface is an essential part to investigate the fundamental quantum phenomena. Experimentally, previous studies on graphene/superconductor devices were usually prepared by the evaporation of superconducting materials over graphene.^[^ ^8^, ^9^, ^10^, ^11^ ^]^ However, it is inevitable to create defects and contaminants at contact interface, and even it is difficult to induce superconductivity coupling with graphene by proximity effect.^[^ ^12^ ^]^ In addition, with the rise of van der Waals heterostructure, another technique of transferring and stacking 2D materials makes a compelling route to explore the proximity effect in hybrid graphene–superconductor devices. Whereas this transfer method depends on advanced techniques to produce sharp and clean interfaces, the devices also undergo weak interfacial interaction and interface contaminants leading to largely degrade device performance.^[^ ^5^, ^13^ ^]^

On the other hand, understanding the scattering processes in 2D materials and heterostructures is fundamentally and technologically important.^[^ ^3^, ^14^ ^]^ By creating atomically thin graphene p‐n junction, various peculiar charge scattering characteristics for massless Dirac fermions, such as Klein tunnelling, Veselago lensing, and Berry phase switch, have been demonstrated experimentally.^[^ ^15^, ^16^, ^17^, ^18^ ^]^ In previous experimental studies, different nanofabricated approaches are used for tackling the resonant scattering potential in graphene, such as etching antidots, substrate engineering, 1D electron guide, ballistic cavities, and depositing periodic gates.^[^ ^19^, ^20^, ^21^, ^22^, ^23^, ^24^ ^]^ However, above nanopatterning procedures inevitably induce extra damage and reduce sample mobility by disorder. Additionally, due to the depletion at boundaries, a well‐defined scattering potential is hard to be realized resulting in a significant distortion of the electron cyclotron orbits.^[^ ^25^ ^]^ So far, these studies of transport in graphene subjected to scattering potentials have been limited to the nanofabrication techniques. The proximity effect induced scattering potential in superconductor/graphene heterostructure junction interface has not been explored.

Recently, we have developed a chemical vapor position (CVD) method to synthesize a series of high‐quality ultrathin transition metal carbides (TMCs) crystals, including Mo_2_C, TaC, and WC.^[^ ^26^, ^27^ ^]^ In α‐Mo_2_C crystals with excellent thermal and chemical stability, the 2D superconductivity has been demonstrated.^[^ ^26^ ^]^ For 2D heterostructures, we also have developed a two‐step CVD approach to fabricate high‐quality graphene/Mo_2_C vertical heterostructures with the double layers of Cu/Mo foils as growth substrates. Employing this approach, a high‐quality graphene/2D superconductor vertical heterostructure with uniform well‐aligned lattice orientation and strong interface coupling has been obtained.^[^ ^28^ ^]^ Compared with the van der Waals heterostructures by conventional transfer and stacking method, this directly grown 2D superconductor/graphene heterostructure has a clean and uniform interface, offering an interesting platform to explore the effect of interface scattering on the transport phenomena.

In this study, we investigate the low‐temperature transport properties of directly grown graphene/Mo_2_C vertical heterostructure devices. By fabricating graphene cross junction with heterostructure located in the central region, we are able to induce an electrostatic potential well to probe charger scattering in graphene. The magnetoresistance curves show a series of resonance‐like peaks at low fields. The temperature and gate voltage‐dependent magnetoresistance behavior are in quantitative agreement with geometric resonance scenario in which the cyclotron radius of incident electron is compatible with effective central scattering boundary. Moreover, the amplitude of magnetoresistance resonance peaks below the superconducting transition temperature *T*_*c*_ of Mo_2_C exhibit a different temperature dependence from the normal state, indicating that the presence of proximity‐induced superconducting gap can effectively modify the confinement strength of the potential well in heterostructure. Our results show that the vertical graphene/Mo_2_C heterostructure exhibit a clean and strong coupling interface for the study of the interplay between Dirac‐like quasiparticles and superconductivity.

## 2 Results and Discussion

### 2.1 Graphene/Mo_2_C Heterostructure Characterization and Device Measurement Configuration

**Figure 1:**
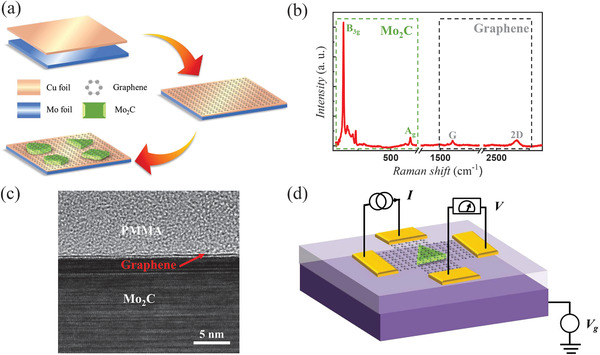
a) Schematic illustrating the growth procedure of graphene/Mo_2_C vertical heterostructures by CVD method. b) Raman spectra of as‐fabricated heterostructures showing characteristic peaks from graphene and Mo_2_C. c) The high resolution cross‐section transmission electron microscopy image of the heterostructures, confirming the high quality of the interface. d) Schematic of the graphene/Mo_2_C heterostructure cross junction device geometry and measurement configuration. A triangular shaped Mo_2_C crystal is located near the central region of the junction.

Ambient‐pressure CVD growth of graphene/Mo_2_C heterostructure and device fabrication process is schematically depicted in **Figure** **1**a. Figure 1b and Figure S1, Supporting Information, show the Raman spectroscopy results, indicating that graphene in such 2D graphene/Mo_2_C vertical heterostructures has very high quality due to the unchanged reaction atmosphere and there is strongly coupled interface between graphene and 2D Mo_2_C crystal. Besides, the high crystalline quality of 2D Mo_2_C can be confirmed by HAADF‐STEM characterization; there is no visible defect or disorder in a large of ≈25 × 25 *nm* ^2^ region (Figure S2, Supporting Information). It is worth noting that graphene is a uniform monolayer film across the whole sample, which includes the part in the heterostructure and the others beyond (Figure S3, Supporting Information). In Figure S4, Supporting Information, it can be found that these heterostructure regions are very smooth and uniform; the thickness of 2D Mo_2_C crystals are 5–20 nm. Cross‐sectional high‐resolution transmission electron microscopy (HRTEM) was performed to identify the sharp and uniform interface for our graphene/Mo_2_C heterostructures, as shown in Figure 1c.

In order to study the effect of Mo_2_C crystal as a scattering potential well on the charge transport properties of graphene, we fabricated a four‐terminal graphene cross junction with Mo_2_C in the central, as illustrated schematically in Figure 1d. The widths of the cross junctions range from 1 to 3 μm, which are close to the size of central Mo_2_C crystals. A typical measurement configuration for the cross junction device is manifested in Figure 1d. The resistance is measured by applying current through two adjacent contacts, while measuring the voltage drop between other two contacts. We also prepared the stacked Mo_2_C/graphene devices by using conventional transfer method for comparison. The device fabrication process is schematically depicted in Figure S5, Supporting Information. However, there are many disadvantages using the transfer method. We can clearly see that the graphene has inevitable wrinkles and cracks (Figure S6, Supporting Information). On the other hand, by two‐step transfer processes, it is unavoidable to be affected by residual photoresistance and other contaminants, which could induce disorder in devices and thereby largely degrade its transport properties.

### 2.2 Graphene/Mo_2_C Heterostructures Cross Junction Device and Resonance Magnetoresistance Peaks

**Figure 2:**
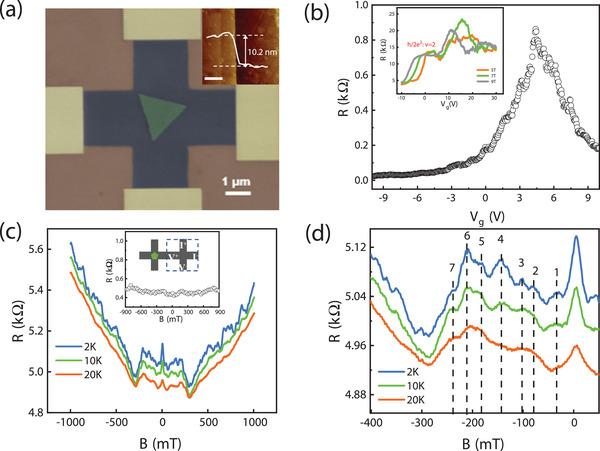
a) False‐colored scanning electron microscope image of a typical graphene/Mo_2_C heterostructure device. The scale bar is 1 µm. Inset: AFM height profile across the edge of heterostructure in the central region of the junction (the scale bar is 0.5 µm). The thickness of the Mo_2_C is about 10.2 nm. b) Transfer characteristics of a graphene/Mo_2_C cross junction device. Inset: the two‐terminal resistance as a function of backgate voltage measured at *T* = 1.9 K under high magnetic fields. The observation of quantum Hall plateaus indicates high electrical quality of our heterostructure device. c) Magnetoresistance traces of graphene/Mo_2_C cross junction for selected temperatures, exhibiting a prominent hump and peak features at low fields. Inset: magnetoresistance of the pristine graphene cross junction in the absence of Mo_2_C crystal for comparison. d) The expanded plot of low‐field magnetoresistance between ‐0.4 and 0 T, exhibiting multiple resonance‐like peaks. The dashed line arrows indicate the magnetic field positions of these peaks, labeled with numbers.

**Figure** **2**a displays a false‐colored scanning electron microscopy (SEM) image of a typical cross junction device. The triangle shape of Mo_2_C with length scale of 1.8 μm is located in the center region of cross junction. The inset of Figure 2a shows corresponding atomic force microscope (AFM) images along the edge of central heterostructure part, showing uniform thicknesses of ≈10.2 nm of Mo_2_C crystal. Figure 2b presents a typical graphene/Mo_2_C heterostructure cross junction resistance as a function of backgate voltage obtained at zero magnetic field. The device exhibits p‐type transfer characteristics with the Dirac point located at around *V*_*bg*_ ≈5 V. The inset of Figure 2b shows the two‐terminal resistance as a function of backgate voltage measured at *T* = 1.9 K under high magnetic fields. The observation of quantum Hall plateaus also confirms that our direct growth method of graphene/Mo_2_C heterostructures have high quality. By applying the gate voltage, we can easily change the carrier density of graphene, which is an advantage for changing the Fermi wavelength of electron in graphene that can help to study the effects related to typical length scale of our device.

Figure 2c presents typical magnetoresistance curves *R*(*B*) of graphene/Mo_2_C heterostructure devices for various temperatures in the magnetic field range from ‐1 to 1 T. In order to characterize the transport through the pristine and heterostructured graphene in the cross‐junction region for comparison, we also fabricated a multi‐terminal Hall bar devices, as illustrated in the inset of Figure 2c. The pristine monolayer graphene cross junction shows a weak magnetic field dependence of the resistance, in agreement with the previous studies.^[^ ^29^, ^30^ ^]^ By contrast, the heterostructure device in the presence of central Mo_2_C crystal displays a broad resistance bump structure, showing an increase of resistance below ±0.25 T. Another feature of the heterostructure device is the appearance of pronounced magnetoresistance oscillations at low fields. Figure 2d shows zoom‐in of the magnetoresistance in a low magnetic field range (‐400–50 mT). At lowest temperature of *T*= 2 K, a series of resonance‐like magnetoresistance peaks can be clearly identified, superimposed on the magnetoresistance traces. These resistance peaks that appear at the specific magnetic fields are reproducible and stable, located symmetrically with respect to the zero field (Figure S7, Supporting Information). As the temperature is increased, the amplitude of the magnetoresistance fluctuations is reduced and gradually smeared out, while the positions of the resistance peaks remain nearly unchanged. These peak positions B* are indicated by the dashed line labeled with numbers as shown in Figure 2d. In contrast to the magnetoresistance behavior of heterostructure devices, the oscillating feature is clearly absent in pristine graphene devices. These observations suggest that the presence of Mo_2_C crystal in heterostructure devices has an important effect on low‐field magnetoresponse.

### 2.3 Potential Well Formation and Geometric Resonance

**Figure 3:**
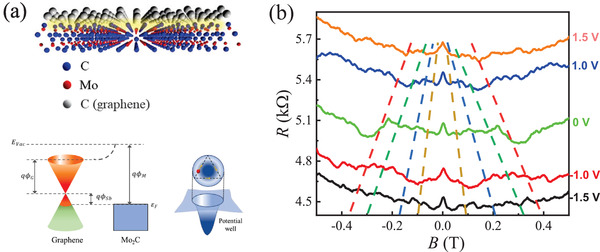
a) Top panel: schematic atomic structure of vertical graphene/Mo_2_C heterostructure. Bottom panel: energy band diagram of the graphene/Mo_2_C heterostructure. The local potential well is formed in the heterostructure region due to the band alignment between Mo_2_C and graphene. b) Low‐field magnetoresistance *R*(*B*) of heterostructure device at different values of applied gate voltages, taken at *T* = 2 K. As we reduce the carrier density by increasing the gate voltage, the field positions of *R*(*B*) peaks move to the lower fields. The dashed lines are guides to the eye.

Considering graphene/Mo_2_C vertical heterostructures in the central junction region, here we propose that the observed magnetoresistance features can be explained in a geometric resonance scenario. In such cross junction configuration, central heterostructures introduce a confining potential for charge carriers in graphene. The cross‐sectional crystalline structure and energy band diagram of vertical graphene/Mo_2_C heterostructures are illustrated in **Figure** **3**a. When 2D Mo_2_C and graphene are brought into contact, the equilibrium state is reached at the interface in which the alignment of the Fermi levels is achieved.^[^ ^31^ ^]^ Considering the work function difference of graphene (≈4.5 eV) and Mo_2_C (≈4.75 eV),^[^ ^32^, ^33^, ^34^ ^]^ charge transfer from graphene causes hole doping with the Fermi level displaced below the Dirac point, which is consistent with the observed p‐type transport characteristics. As a consequence, an electrostatic scattering potential well can be formed in the central heterostructure region. Similar potential barrier has been reported in other graphene heterostructures.^[^ ^35^, ^36^ ^]^ The bottom panel of Figure 3a depicted a schematic illustration of the boundary scattering processes in a potential well, being analogous to the geometric resonance for electrons in cavity nanostructures.^[^ ^37^, ^38^, ^39^ ^]^ As a perpendicular magnetic field is applied, injected electrons move along the cyclotron orbits entering into the potential well imposed by the heterostructure, leading to additional charge carriers scattering at the boundaries. When the magnetic field is swept to meet resonance condition, the cyclotron orbits of electrons form a stable closed orbit which is confined within the potential well, giving rise to the magnetoresistance peak at certain magnetic fields. Similar commensurability magnetoresistance oscillations have been also observed in 2D electron gas subjected to a periodically modulated potential.^[^ ^19^, ^22^, ^23^, ^24^ ^]^ According to the description of classical electron cyclotron motion radius

(1) $$R_{c}=\frac{\hbar k_{F}}{eB^{*}}$$

here $k_{F}=\sqrt{2\pi n_{s}}$ is Fermi wave vector, ℏ is Planck's constant, *n*_*s*_ is the carrier density of the graphene. Thus, the resonance condition can be achieved by varying the magnetic field or carrier density.

To further investigate geometric resonance in graphene/Mo_2_C heterostructure based junction devices, we carried out the gate voltage‐dependent magnetoresistance measurements. Figure 3b shows a series of magnetoresistance traces taken at different gate voltages from ‐1.5 to 1.5 V as indicated. Pronounced resonance resistance peaks appear symmetrically with respect to zero magnetic field. As shown in Figure 2b, the Dirac point of heterostructure device locates at positive gate voltage (V_*Dirac*_ ≈5 V). Thus, with increasing gate voltage in the hole side, the carrier density is tuned to decrease. When the gate voltage is increased approaching the Dirac point, it can be found that the resonance peaks gradually move to lower magnetic fields, as indicated by the dashed lines. Such evolution is expected from Equation (1) since the field position for given geometric resonance condition becomes smaller with decreasing carrier density. In Figure S8, Supporting Information, we present magnetoresistance curves *R*(*B*) of stacked Mo_2_C/graphene devices using transfer method taken at different temperatures and gate voltages, respectively. In contrast to the magnetoresistance behavior of our grown directly Mo_2_C/graphene heterostructure devices, no resistance bump structure and oscillations were observed in stacked devices. The gate voltage evolution of magnetoresistance is also not regular. No noticeable feature of resonant scattering was observed. These comparative results further support that the presence of clean and strong coupling interface in our directly grown Mo_2_C/graphene heterostructures can effectively modify the potential well, being responsible for the observed geometric resonance.

### 2.4 Geometric Calculation of Stable Cyclotron Orbits

**Figure 4:**
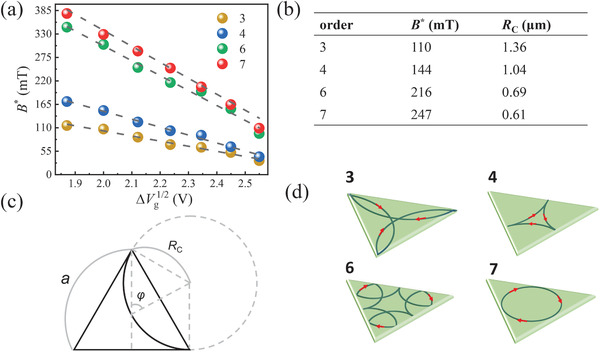
a) The field position B* corresponding to magnetoresistance resonance peaks 3, 4, 6, 7 extracted from Figure 3a as a function of the square root of the gate voltage. The dashed lines are linear fits, confirming the geometric resonance nature of these peaks. b) The table shows the field position B* and electron cyclotron motion effective radius *R*_*c*_ corresponding to order 3, 4, 6, 7 magnetoresistance resonance peaks. c) Geometry demonstration of trajectory of the electron in potential well. d) Schematic sketch of the stable‐orbit electron trajectories within triangle potential well, corresponding to order 3, 4, 6, 7 magnetoresistance resonance peaks, respectively.

We next carry a quantitative analysis of magnetoresistance resonance peaks. For monolayer graphene, the carrier density near the Dirac point is induced by gate voltage with linear dependence *n*∝*V*_*g*_. Therefore, we can quantitatively evaluate the B* by performing a square root dependence of the gate voltage. For clarity, the gate voltage at the Dirac point has been shifted to zero. **Figure** **4**a plots the field positions B* of order 3, 4, 6, 7 magnetoresistance resonance peak as a function of Δ$V_{g}^{1/2}$. The dashed lines show the results of linear fitting. It can be clearly seen that the values of B* scale with $V_{g}^{1/2}$, which is consistent with the picture of geometric resonance in a hard‐wall potential well of 2D graphene/Mo_2_C heterostructure cross junction.

Here, we pick the order 3, 4, 6, 7 megnetoresistance resonance‐like peaks in Figure 2d as examples to quantitatively analyze the formation of stable cyclotron orbits of electrons in the hard‐wall potential well. The geometric parameters of the device we measured are as follows: The height of the central Mo_2_C is 1.8 µm and the area is close to 2.5 µm^2^, here approximately considered as an equilateral triangle model. For the third‐order resonant peak, the characteristic magnetic field B* is 0.11 T. According to the Equation (1), we can estimate the corresponding radius *R*_*c*_ is 1.36 µm as shown in table in Figure 4b and further circumference is 8.54 µm. Combining with the geometric relations as demonstrated in Figure 4c, we are able to infer the trajectory of the electron in the potential well based on the total length of the circle corresponding to the trajectory as sketched in Figure 4d. The trajectory of other order magnetoresistance resonance peaks can also be deduced by the above analogy, which is consistent with geometric calculation results in Figure 4b.

### 2.5 Superconducting Proximity Effect on the Magnetoresistance Fluctuation

**Figure 5:**
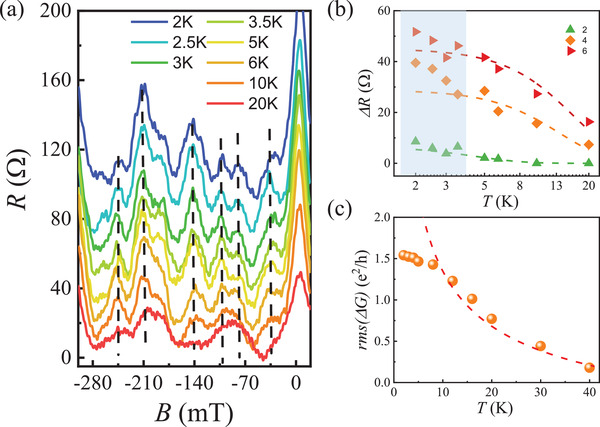
a) Temperature evolution of magnetoresistance peaks in the range between 2 and 20 K, obtained by subtracting smooth background. The curves are offset vertically for clarity. b) Amplitude of the different orders of magnetoresistance peaks ΔR as a function of temperature. The dashed curves are the fits by the thermal smearing model. c) Root‐mean‐square of the magnetoconductance fluctuation rms(Δ*G*) dependence of temperature. The dashed line corresponds to *T* ^−0.5^ power‐law dependence.

Finally, we turn our attention to the effect of superconductivity on the magnetotransport properties of graphene/Mo_2_C heterostructure junction. **Figure** **5**a presents a detailed temperature dependence of the magnetoresistance from 2 to 20 K, subtracting a polynomial resistance background. For clarity, the individual traces are shifted vertically respectively at 2 K. Upon increasing the temperature, the series of resonance peaks are smeared out gradually. It can be clearly seen that the amplitude of the low‐field oscillations decreases with increasing temperature. In Figure 5b, we plot the extracted oscillation amplitude for different orders as a function of temperature. Following previous studies of 2D electron gas subjected to a periodic potential, the increasing temperature smears the cyclotron radius of geometric resonance manifesting as a damping of magnetoresistance oscillations. It has been established that the amplitude of magnetoresistance can be described by^[^ ^25^, ^40^ ^]^

(2) $$\begin{array}{c} \frac{\Delta \rho_{xx}}{\rho_{0}}={\left[\frac{eV_{0}}{\varepsilon_{F}}\right]}^{2}\left[\frac{l^{2}}{aR_{c}}\right]\cos^{2}\left[\alpha \varepsilon_{F}^{1/2}-\frac{\pi }{4}\right]\times \left(\frac{T}{T_{a}\left(B\right)}\right)\mathrm{csch}\left[\frac{T}{T_{a}\left(B\right)}\right] \end{array}$$

where *V*_0_ is the amplitude of the potential, ρ_0_ is the resistivity in zero magnetic field, α = 2π(2m*)^1/2^/*eBa*, and *k*_*B*_ *T*_*a*_(*B*) = ℏω_*c*_ *k*_*F*_ *a*/4π^2^. The fitting results are shown as gray dashed lines in Figure 5b. It can be seen that the data at high temperatures show good agreement with Equation (2), revealing a character of classical electron cyclotron motion. However, the oscillation amplitude at low temperatures exhibits a noticeable upturn below the superconducting transition temperature *T*_*c*_ ≈ 4 K of 2D Mo_2_C,^[^ ^26^ ^]^ highlighted by the blue region in Figure 5b. Such significant deviation near *T*_*c*_ suggests a connection with superconductivity which is developed in Mo_2_C. For our graphene/Mo_2_C heterostructures, superconductivity would expect to be induced in graphene through strong couple interface. As a result, a proximity‐induced superconducting gap could be formed in the interface region, which has been identified in hybrid graphene‐superconductor structures by using scanning tunnelling microscopy.^[^ ^12^, ^41^ ^]^ Comparing with the situation in the normal state, the presence of proximity superconductivity gap may modify the confinement strength of the potential well underneath the Mo_2_C in the junction region. Therefore, the different temperature dependence of the magnitude of resonance peaks provides the evidence for superconducting modification effect.

We further take the analysis of overall conductance fluctuations averaged over magnetic fields, which can provide additional information on charge carrier scattering and dephasing mechanisms. In order to quantify the conductance fluctuations, we subtract the slowly varying background conductance. Figure 5c shows the extracted conductance fluctuations as a function of temperature. The amplitude of the fluctuations decreases with increasing temperature, in agreement with the universal conductance fluctuations behavior due to mesoscopic electronic interference.^[^ ^42^ ^]^ For a 2D graphene, the amplitude of the fluctuation scales with the phase coherent length, which is usually a power law of the form ∼ *T* ^−α^.^[^ ^43^ ^]^ In the high‐temperature regime, it is seen that the data follow the power law behavior rms(δ*G*) ∼ *T* ^−α^ with α ≈ 0.5, which is related to the dephasing mechanism of Coulomb interaction between charge carriers. As the temperature is further lowered, the growth of amplitude deviates from the ∼ *T* ^−0.5^ dependence. The characteristic temperature (≈ 4–5 K) is comparable with the *T*_*c*_ value of Mo_2_C crystals, suggesting that the fluctuations are strongly influenced by the induced superconductivity in heterostructure. Previous experimental and theoretical studies in mesoscopic normal metal/superconductor systems have found that the proximity effect would modulate the conductance fluctuations, which have been attributed to the presence of the Andreev reflection at the interface.^[^ ^42^, ^44^, ^45^ ^]^ However, the magnitude of the fluctuations and its relation to superconductivity and mesoscopic structures remain a controversy.^[^ ^44^, ^45^, ^46^, ^47^ ^]^ Our graphene/Mo_2_C heterostructure with clean and strong coupling interface may provide an ideal platform for studying the relationship between conductance fluctuations and proximity effect in graphene.

## 3 Conclusion

In conclusion, we have realized high‐quality hybrid graphene‐superconductor devices with clean and strong coupling interface based on directly grown graphene/2D superconducting Mo_2_C vertical heterostructure. We observe a series of resonance‐like magnetoresistance peaks at low temperature. The evolution of the magnetotransport features with temperature and gate voltage can be explained in terms of geometric resonance by the interaction of the carriers with the boundaries of the potential well formed at the graphene/Mo_2_C interface. Further analysis of the amplitude of magnetoresistance resonance peaks and magnetoconductance fluctuations reveals the different temperature‐dependent behavior between the normal and superconducting state, indicating that the confinement strength of the potential well can be effectively modified through superconducting proximity effect in heterostructure. Our findings open the door to exploration of directly grown graphene/2D superconductor vertical heterostructures as the ideal platform for investigating the interplay of massless Dirac fermion and superconductivity.

## 4 Experimental Section

### Growth of Graphene/Mo_2_C Vertical Heterostructures

Ambient‐pressure CVD growth of graphene/Mo_2_C heterostructure followed these steps. A Cu foil was cut into pieces and placed on the top of Mo foil which had the same size of the Cu foil. Then, they were placed in a quartz tube as the growth substrate of graphene/Mo_2_C heterostructure. First, using methane (CH_4_, 1.2 sccm) as the carbon precursor and under H_2_(200 sccm)/Ar(500 sccm) as the carrier gas, we set growth temperature of 1070 ° C in a horizontal tube furnace. After 30 min, the graphene has been formed. The Cu/Mo substrate was heated to 1090 ° C to grow 2D Mo_2_C. At the present temperature, Cu foil started to melt and covered the entire Mo substrate. The methane through graphene further reacted with Mo to form α‐Mo_2_C, which was underneath the graphene on the liquid Cu/Mo substrate. Finally, setting flow rate of methane made the samples cool down to room temperature.

### Structure Characterization of Graphene/Mo_2_C Heterostructure

Graphene/Mo_2_C heterostructure on Cu/Mo substrate was confirmed by Raman spectroscopy with a 632.8 nm laser (Jobin Yvon LabRAM HR800). The cross‐sectional HRTEM image of graphene/Mo_2_C heterostructure was taken by TEM with a voltage of 300 kV (FEI Titan3 G2 60‐300), and cross‐sectional sample was processed by Focused Ion Beam with Ga source (Zeiss Orion NanoFab).

### Device Fabrication and Transport Measurement

The graphene/Mo_2_C heterostructures were transferred onto SiO_2_/Si substrates by means of conventional poly(methyl methacrylate)‐mediated method.^[^ ^26^ ^]^ After the transfer process, the graphene/Mo_2_C heterostructures crystals were selected and located by optical microscopy. Titanium/gold(5/90 nm) electrodes were patterned on each arm of the cross junction by means of standard electron beam lithography followed by electron beam evaporation of the metals. The electrical transport measurements were performed in a physical property measurement system (Quantum Design DynaCool) from room temperature to 1.8 K. Here, the standard four‐terminal resistance was measured using the low‐frequency lock‐in technique with an excitation current of 1–5 µA at a frequency of 17.77 Hz. The magnetic field was applied perpendicular to the plane of the sample.

### Statistical Analysis

The intensities of Raman data in Figure 1b and Figure S1, Supporting Information, were normalized. The statistical analysis was performed using OriginPro 2016 (Origin‐Lab Corporation).

## Conflict of Interest

The authors declare no conflict of interest.

## Supporting information

Supporting Information

## Data Availability

The data that support the findings of this study are available from the corresponding author upon reasonable request.
