# Supplementary material for: Resonant Scattering in Proximity‐Coupled Graphene/Superconducting Mo2C Heterostructures
Source: Adv Sci (Weinh). 2022 May 23;9(21):2201343. doi: 10.1002/advs.202201343 (PMC9313478; doi:10.1002/advs.202201343)
Supplement: Supplementary file 1 — Supporting Information [file ADVS-9-2201343-s001.pdf]

ADVANCED SCIENCE
Open Access

Supporting Information

for *Adv. Sci.*, DOI 10.1002/advs.202201343

Resonant Scattering in Proximity-Coupled Graphene/Superconducting Mo_2C Heterostructures

Meng Hao, Chuan Xu, Cheng Wang, Zhen Liu, Su Sun, Zhibo Liu, Hui-Ming Cheng, Wencai Ren and Ning Kang**

Supplementary Material: Resonant Scattering in Proximity-Coupled Graphene/ Superconducting Mo_2C Heterostructures

Meng Hao Chuan Xu* Cheng Wang* Zhen Liu Su Sun Zhibo Liu Hui-Ming Cheng Wencai Ren Ning Kang*

Meng Hao, Cheng Wang, Zhen Liu, Ning Kang
Key Laboratory for the Physics and Chemistry of Nanodevices and School of Electronics, Peking University, Beijing 100871, China
Email Address: nkang@pku.edu.cn

Chuan Xu, Su Sun, Zhibo Liu, Hui-Ming Cheng, Wencai Ren
Shenyang National Laboratory for Materials Science, Institute of Metal Research, Chinese Academy of Sciences, Shenyang 110016, China
Email Address: wcren@imr.ac.cn

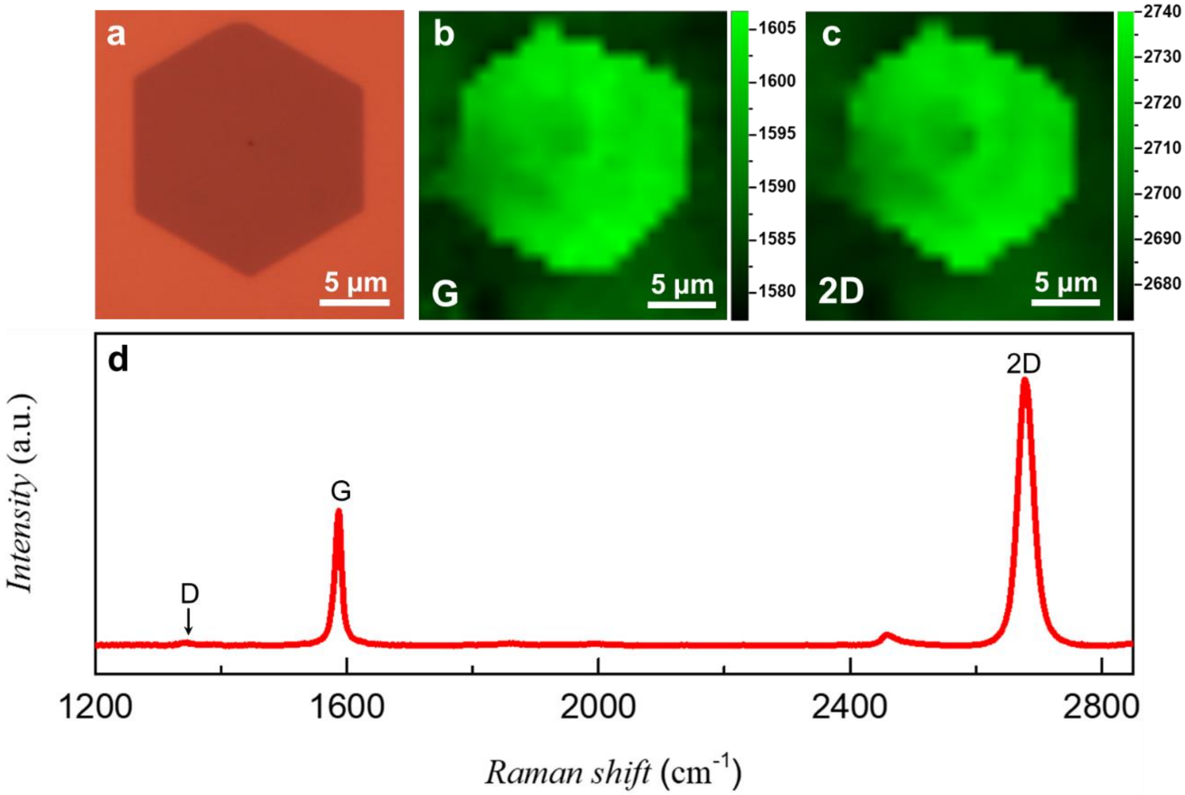

Figure S1: (a) Optical images of a graphene/2D Mo_2C crystal heterostructure grown on SiO_2/Si substrate. (b), (c), The corresponding Raman peak position maps of G (b) and 2D (c) of graphene in a. (d) Raman spectrum of graphene transferred on SiO_2 (290 nm)/Si substrate beyond heterostructure region.

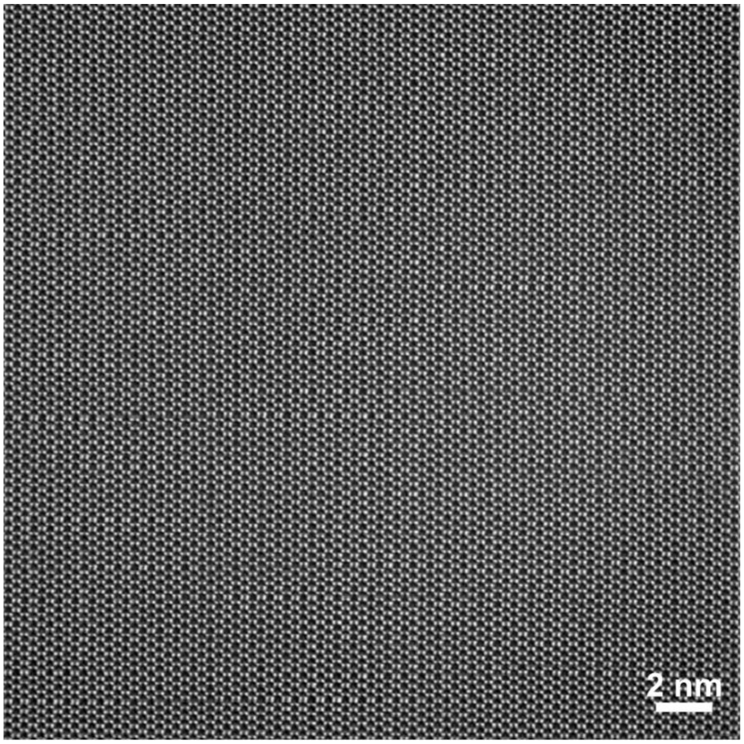

Figure S2: Atomic-level HAADF STEM image of a 2D Mo_2C crystal in heterostructure taken from a large of 25×25 nm^2 region. No defect or disorder can be observed in such large area, which means 2D Mo_2C crystal has a high crystalline quality.

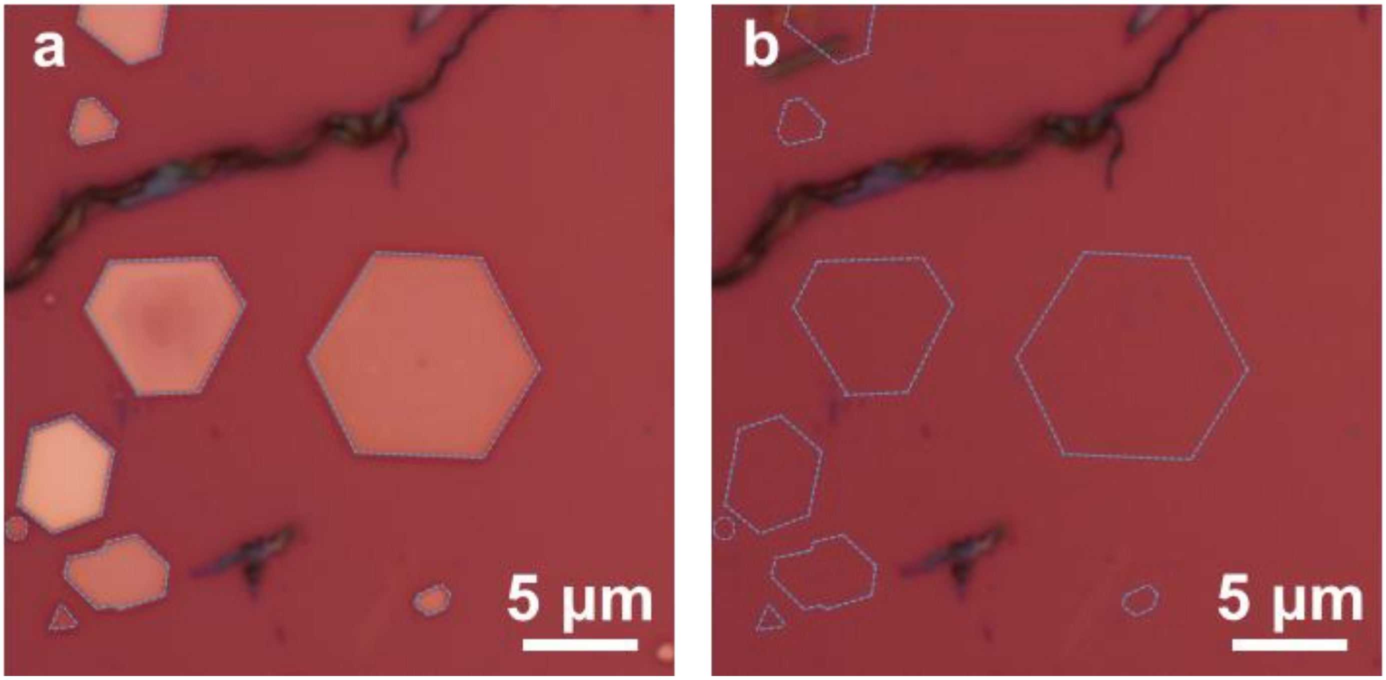

Figure S3: (a) Optical images of as-transferred graphene/2D Mo_2C crystal heterostructures on SiO_2/Si substrate. (b) The corresponding optical images of the sample in a after removing 2D Mo_2C crystals by using 0.2 M $(NH_4)_2S_2O_8$ solution.

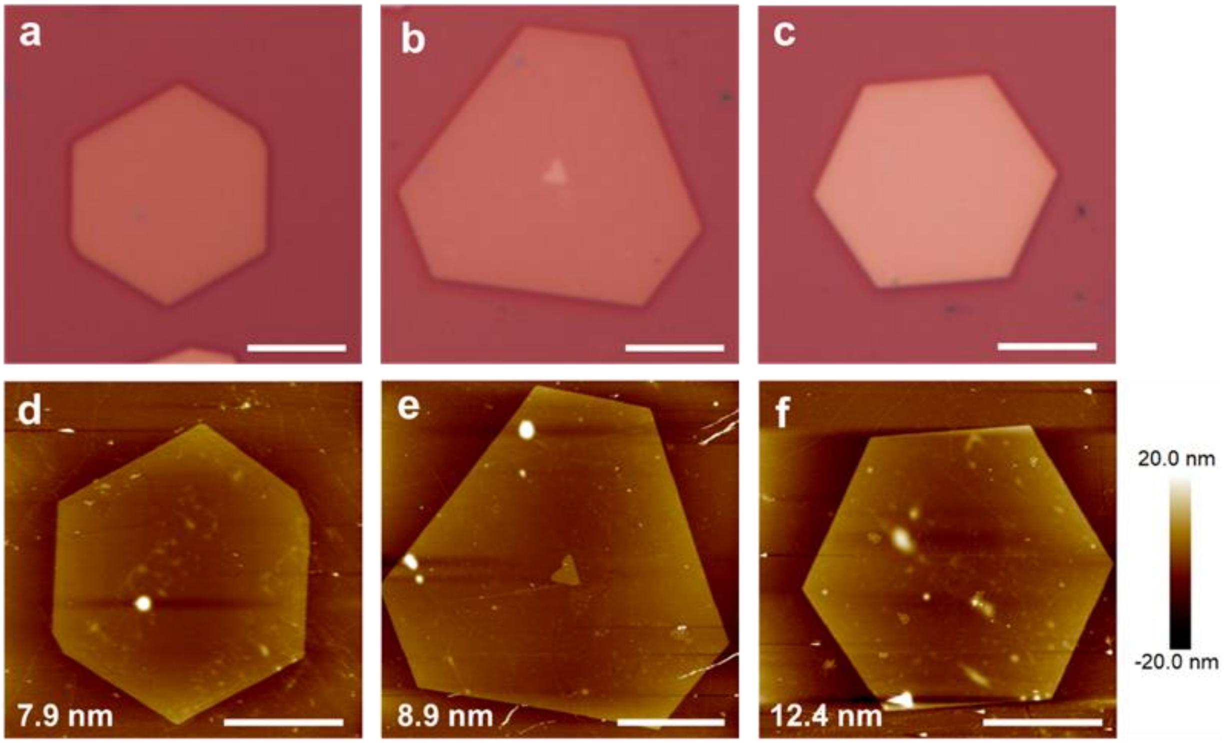

Figure S4: Graphene/2D Mo_2C crystal heterostructures transferred on SiO_2 (290 nm)/Si substrates. (a-c) Optical images of the heterostructures with three different hexagonal 2D Mo_2C crystals. (d-f) The corresponding AFM images of graphene/2D Mo_2C crystal heterostructures with the thickness of 7.9 nm (d), 8.9 nm (e) and 12.4 nm (f). All scales are 5 μm in a-f. It can be found that the crystals are uniform and smooth.

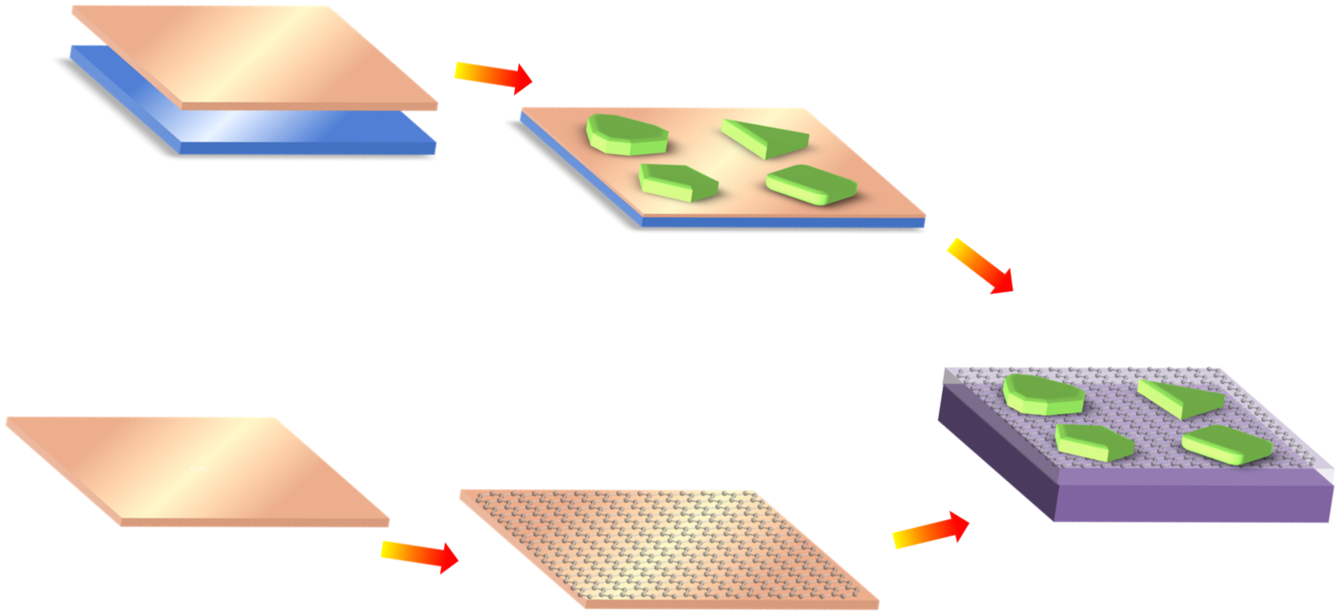

Figure S5: Schematic illustrating the CVD growth and two step transfer procedures of stacked Mo_2C/graphene heterostructures.

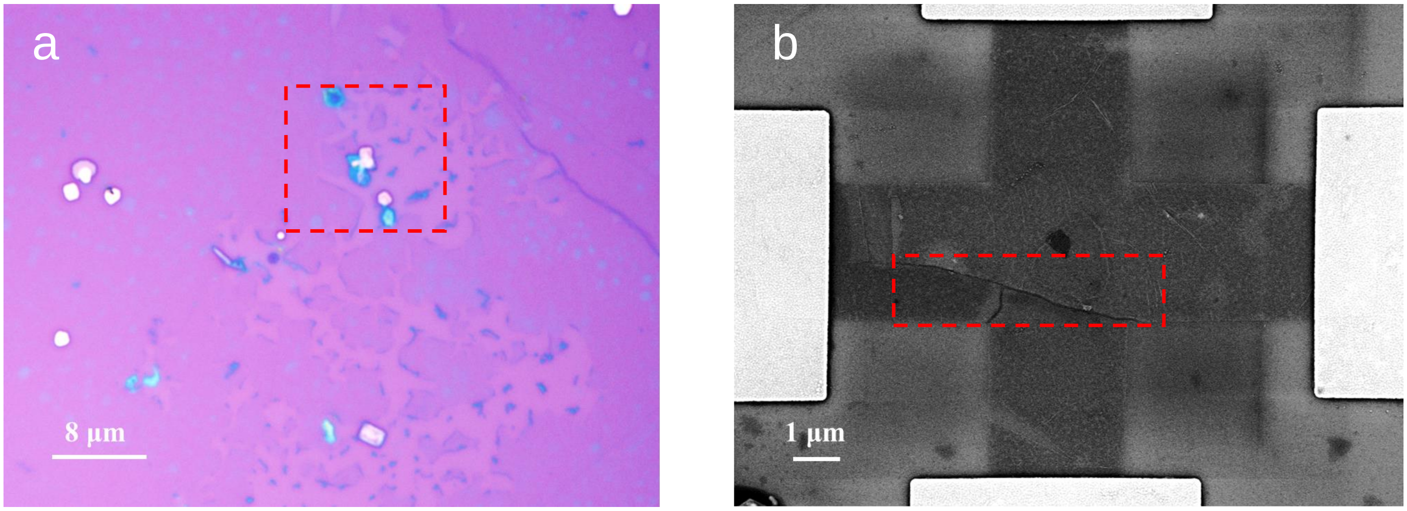

Figure S6: (a) The optical image of stacked Mo_2C/graphene samples on SiO_2/Si substrate. (b) The scanning electron microscope (SEM) image of a typical stacked Mo_2C/graphene heterostructure device, cracks in graphene as shown in red dotted box.

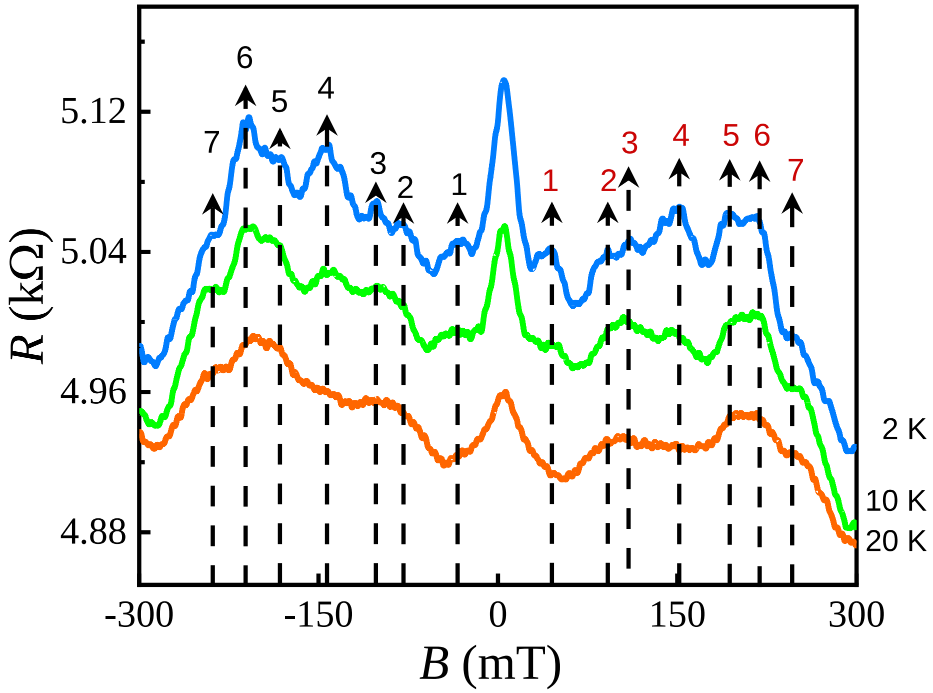

Figure S7: The expanded plot of low-field magnetoresistance between -0.3 and 0.3 T, exhibiting multiple resonance-like peaks. The dashed line arrows indicate the magnetic field positions of these peaks, which locate symmetrically at both positive and negative fields.

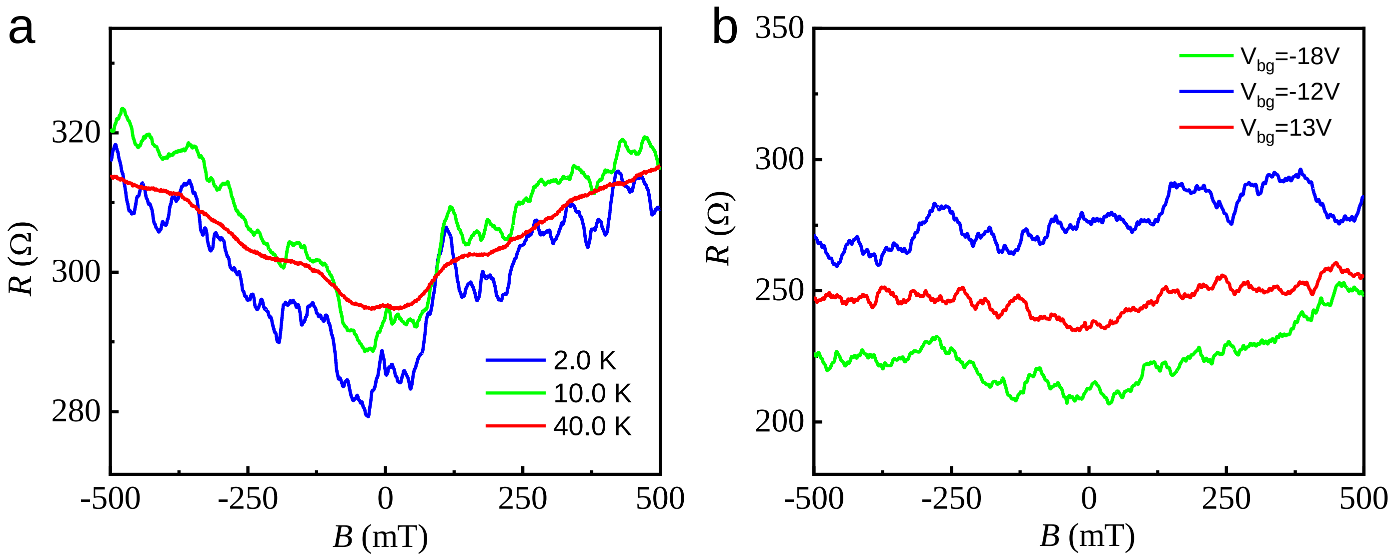

Figure S8: (a) Magnetoresistance traces of stacked Mo_2C/graphene devices for selected temperatures at low fields. (b) Low-field magnetoresistance *R(B)* at different values of applied gate voltages, taken at $T = 2$ K.
